# Detecting sleep outside the clinic using wearable heart rate devices

**DOI:** 10.1038/s41598-022-11792-7

**Published:** 2022-05-13

**Authors:** Ignacio Perez-Pozuelo, Marius Posa, Dimitris Spathis, Kate Westgate, Nicholas Wareham, Cecilia Mascolo, Søren Brage, Joao Palotti

**Affiliations:** 1 MRC Epidemiology Unit, School of Clinical Medicine, University of Cambridge, Cambridge, UK; 2 The Alan Turing Institute, London, UK; 3 School of Clinical Medicine, University of Cambridge, Cambridge, UK; 4 Department of Computer Science and Technology, University of Cambridge, Cambridge, UK; 5 Qatar Computing Research Institute, Hamad Bin Khalifa University, Doha, Qatar

**Keywords:** Biomarkers, Diagnosis, Disease prevention, Quality of life, Medical research

## Abstract

The adoption of multisensor wearables presents the opportunity of longitudinal monitoring of sleep in large populations. Personalized yet device-agnostic algorithms can sidestep laborious human annotations and objectify cross-cohort comparisons. We developed and tested a heart rate-based algorithm that captures inter- and intra-individual sleep differences in free-living conditions and does not require human input. We evaluated it on four study cohorts using different research- and consumer-grade devices for over 2000 nights. Recording periods included both 24 h free-living and conventional lab-based night-only data. We compared our optimized method against polysomnography, sleep diaries and sleep periods produced through a state-of-the-art acceleration based method. Against sleep diaries, the algorithm yielded a mean squared error of 0.04–0.06 and a total sleep time (TST) deviation of $-$2.70 (± 5.74) and 12.80 (± 3.89) minutes, respectively. When evaluated with PSG lab studies, the MSE ranged between 0.06 and 0.11 yielding a time deviation between $-$29.07 and $-$55.04 minutes. These results showcase the value of this open-source, device-agnostic algorithm for the reliable inference of sleep in free-living conditions and in the absence of annotations.

## Introduction

Human sleep is a reversible physiological state that is essential for health and performance^1^. Its functions are not fully understood, despite extensive studies on its its influence on energy homeostasis, immune function, cognitive performance and behaviour^2–10^. As such, sleep lies at the cross-roads of multiple research programs in both life sciences and public health. This makes the objective monitoring of sleep crucial for understanding human health. Apart from outright sleep disorders, sleep patterns impact the quality of life and history of common diseases, whether cardiovascular, metabolic or neurodegenerative. The gold-standard method for quantifying sleep quantity and quality is polysomnography (PSG). PSG requires signals from multiple sensors, as well as expert input and is thus limited to the laboratory. This has limited large-scale and long-term population studies. Furthermore, the unfamiliar laboratory environment might not favor the patients’ typical sleep pattern^11^.

Actigraphy is an established and popular alternative to PSG. It originates in early telemetric measurements of motor activity in the 1970s which were used to assess sleep quality^12^. Since then, many studies have assessed actigraphy against PSG^13–15^. The advantage over PSG is that actigraphy, and modern counterpart, accelerometry, require sensors amenable to affordable wrist-worn devices^13,16^. At present, the use of actigraphy in healthy sleepers is approved by both the FDA and recommended by the American Academy of Sleep Medicine (AASM)^14^.

Over the past 30 years, several actigraphy-based algorithms have been developed to detect night-time sleep and wake periods. Some have proved to have strong validity and reliability against PSG^17–22^. These algorithms have been widely adopted and were recently bench-marked against both each other and newer machine learning and deep learning methods^23–26^. Actigraphy often struggles to classify wake events during the sleep period, yielding poor specificity compared to PSG^16,21,23,27,28^. Additionally, actigraphy algorithms have only been optimised to detect sleep at night, as study participants typically spend one or two nights in the laboratory. Using them with data recorded over the entire day is unreliable, even with sleep annotations provided by PSG technicians or the subjects themselves^29,30^. This limitation extended to proprietary algorithms used by commercial wearables. In addition, actigraphy devices do not provide real-time feedback about the user’s sleep, which hinders longitudinal monitoring. The result is that unconventional sleep patterns (eg. due to shift work) have been understudied.

Novel wearables add photoplethysmography (PPG)-derived heart rate to the accelerometry signal. These multimodal devices rely on recent advances in microelectromechanical systems (MEMS) along with improvements in cost, battery capacity, and memory, allowing for higher sensor sampling rates. The widespread adoption of these devices for both research and commercial use promises robust inferences about the user’s sleep/wake periods. To this end, large investments have been made both by companies providing personal health monitoring and through research grants for programs such as “All of US”^31^. The increased attention has made paramount the need for validation against PSG^32^, given some have recently even claimed to label sleep stages^33,34^. Furthermore, incorporating heart rate (HR) and heart rate variability (HRV) connects sleep algorithms directly with the cardiovascular fitness predictors that many studies focus on^24,35,36^.

Whilst valuable, these approaches have limited generalizability due to three reasons: first, lack of domain adaptation between different datasets used to train the models; second, lack of data recorded beyond night-time^37^. Finally, they rely on self-reported sleep (through diaries or questionnaires), which is prone to recall bias^38,39^. Even with regular sleepers taking careful diaries, it can take more than 6 recorded nights to match the diaries with objective labels^40^. This period is close to usual study lengths, which are limited by device battery life. Sidestepping laborious annotations would encourage adoption by more users over a longer time, providing abundant and cheaper data for further improving inference algorithms.

Our sleep detection algorithm leverages heart rate data available from most commercial and research-grade wearable devices. The algorithm is device-agnostic and matches the quantitative sleep/wake inferences offered by previous methods. We validated it against four datasets where heart rate data was recorded together with multiple PSG-grade sensors or actigraphy. As our algorithm does not, unlike machine learning models, require training before deployment, it can run on the device independently of cloud computing. This preserves user privacy, a paramount concern for health data. The algorithm was first developed in a large population (n=193) with about 8 recorded nights accompanied by detailed sleep diaries. This cohort wore a combined heart rate and movement sensor, in addition to a set of 3 accelerometers on both wrists and hip. This provided the additional opportunity to benchmark against previous algorithms relying on accelerometer angle changes. Data from multiple consecutive days and nights facilitated testing for inter- and intra-individual variability (i.e., sleep statistics across the entire cohort or across each participant’s sleep windows). We then assessed our method in a larger, more diverse, open-source dataset (n=1,743), as well as a smaller cohort (n=31) that used a commercial-grade device (Apple Watch) during PSG. Finally, performance in free-living conditions was tested against sleep diaries in a cohort (n=22) wearing accelerometer/heart rate sensor.

## Methods

### Data sources and processing

In this study we used four different data sources with a variety of devices and populations to showcase the performance of our proposed method. Table 1 summarizes the types of wearable devices and ground truth used in each one of the studies. We describe the detailed data processing used in each one of these datasets in the supplementary material. The study was carried out in accordance with relevant guidelines and regulations. Note that MESA^41–43^, Apple Watch PhysioNet^33^ and MMASH^44^ are publicly available datasets (see detailed on supplementary material) while BBVS^45^ is restricted. No identifying participant information was available to the authors of this study.

**Table 1:** Summary of population size and devices used in the different datasets.

Study	# Participants	Sensor type	Wearable device make	PSG	Sleep Diary
*Biobank validation study*	158	Triaxial accelerometer (3) Wearable ECG	AX3, Axivity (Newcastle,UK) Actiheart, CamNtech (Cambridge,UK)		$\checkmark$
*Multi-ethnic study of atherosclerosis*	1154	Actigraphy monitor ECG	Actiwatch Spectrum, Philips Respironics (PA,USA)	$\checkmark$	$\checkmark$
*PhysioNet apple watch*	22	Triaxial accelerometer Heart rate sensor (PPG)	Apple Watch (Series 2,3), Apple (CA, USA)	$\checkmark$	
*Multilevel monitoring of activity and sleep in healthy people*	20	Triaxial accelerometer Heart rate sensor	ActiGraph wGT3X-BT, ActiGraph LLC (FL,USA) Polar H7, Polar Electro Inc (NY,USA)		$\checkmark$

### Algorithm to estimate the sleep window using heart rate

Several challenges must be accounted for when developing a method for the detection of sleep in free-living conditions. First and foremost, most methods derived for sleep-wake classification using wearable devices have been derived on and for use during the night period^17,19,20,23,33^. These approaches were mostly conducted in small studies using concurrent PSG and as such, their application during the full day period greatly compromises the quality of the results. They also tend to be optimized in small, non-diverse populations, comprising their generalizability to other cohorts. Moreover, they tend to be device and make specific, often requiring conversions into arbitrary activity intensity measures or counts. Finally, most algorithms that can be applied during the 24 hour period require sleep diaries or questionnaires for guidance, which are often biased and burdensome to obtain^46^.

Here we introduce a simple approach to estimate sleep window leveraging the HR sensing capabilities that most modern wearables have. One of the major challenges presented by large cohort studies is inter-individual differences. For instance, individuals who are fitter, tend to have lower resting heart rates than those who are not as fit^47^. Hence, an approach that relies on HR signals should not follow a *one size fits all*, but rather adapt to each individuals’ own heart rate profiles. To account for these considerations, we use the empirical cumulative distribution function (ECDF) of each individual’s daily heart rate profile. This function, *F*(*x*), is the probability that for each individual their heart rate takes a value *x* such that:

1 $$\begin{aligned} F(x) = P(X_{i} \le x), \end{aligned}$$

for every sequence $i = 1, ..., n$. Namely, *F*(*x*) is the probability of the event $\{ X_{i} \le x \}$. In this case, *x* is a threshold heart rate value (in beats per minute). To estimate the probability of a given event, we turn to the ratio of such an event given an individual’s daily sample of heart rates. This results in:

2 $$\begin{aligned} {\hat{F}}_{n}(x) = \frac{\text {number of } X_{i} \le x}{\text {total number of observations}} = \frac{\sum _{i = 1}^{n} I(X_{i} \le x)}{n} = \frac{1}{n} \sum _{n}^{i=1}I(X_{i}\le x) \end{aligned}$$

as the estimator of *F*(*x*), that is the ratio of HR less than *x*, where *I*() is the *indicator function*.

Thus, for every *x*, we can use such quantity as an estimator, so the estimator of the cumulative distribution function, *F*(*x*) is $\hat{F}(x)$, which is referred to as the *empirical cumulative distribution function*.

By using the HR cumulative distribution function for each participant and each day of recording, our method accounts for inter- and intra-individual variation. It can adjust to different levels of fitness which often result in different resting HR during sleep^47^. Further, an elevated resting heart rate (RHR) accompanied by a fever is a well-known response to infection^48^, alcohol consumption^49^, stress^50^ and can even be used to monitor influenza-like illness^51^, something that our approach would account for. The method contains no in-built assumption of absolute time for the sleep window, and can therefore be used in night shift-workers and non-monophasic sleepers (those whose have more than one principal sleep windows in a 24-hour period) where the circadian HR rhythm is shifted so that most of the lower HR values still occur during sleep independent of the absolute time window when their sleep takes place. An example of our method applied to a shift worker can be observed in Supplementary Fig. S1.

**Figure 1:**
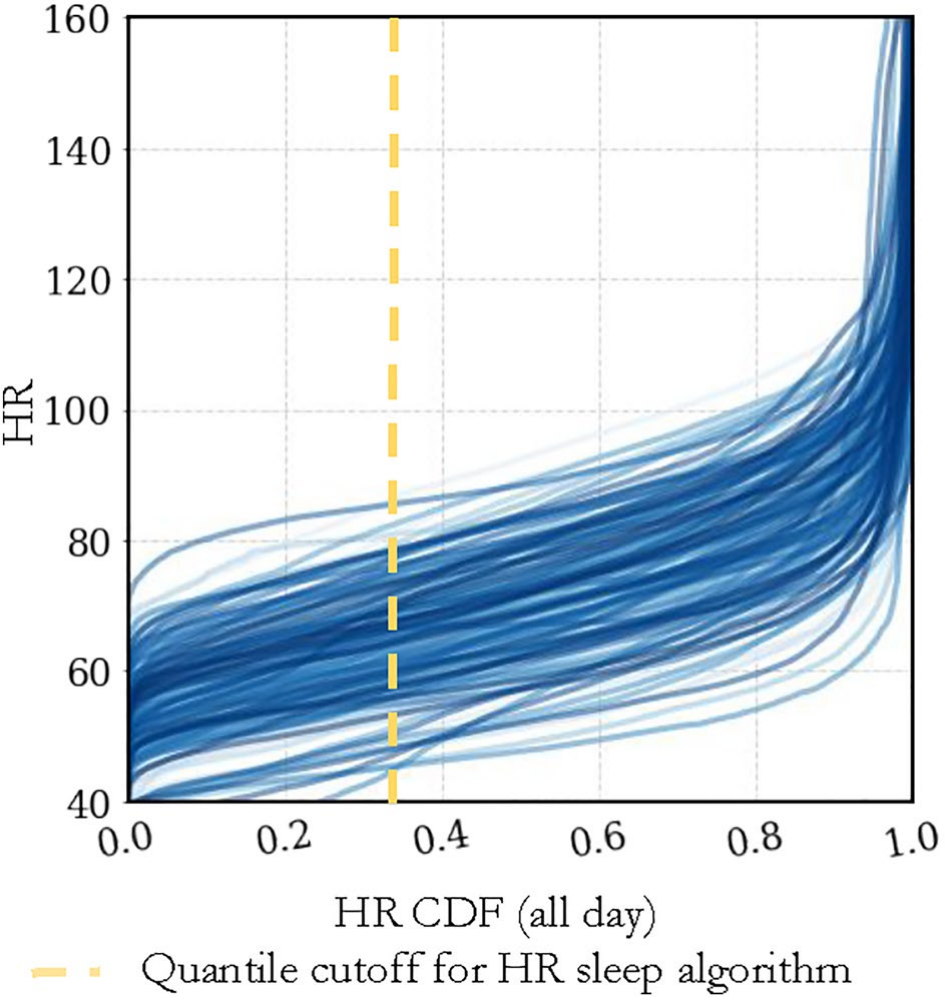
Cumulative distribution function for BBVS heart rates. The figure shows the HR ECDF for the full-day across all participants and all days, where the yellow dotted line shows the 0.35 HR quantile cutoff. Each individual line represents one participant for one day of recording.

The first step of our heart rate sleep algorithm involves pre-processing the time series by assigning binary wake/sleep labels whenever the participant’s heart rate dips above/below a specific **quantile threshold (***Q***)**. The threshold value is calculated from the ECDF over 24-hour windows arbitrarily starting at 15:00 each day. Figure 1 showcases this cutoff for the full BBVS population based on two intervals (full day and from 21:00 to 11:00, a conventional night).

Wake/Sleep labels are then smoothed with a 5-minute rolling median and the length of their sequences is calculated. Sequences of sleep labels that are longer than a **minimum length **(*L*) are extracted and merged with other sleep sequences if their **gap length **(*G*) is smaller than a pre-defined value. We study the behavior of the parameters *Q*, *L* and *G* for each dataset with the goal of finding the best possible combination.

To be eligible as part of the final sleep window, the sleep sequence must not be preceded by more than 90 minutes of wake in the previous 4 hours of recording. The limits of the merged sleep sequences then guide a search (in a window starting 240 minutes before and 60 minutes after) for epochs with high HR volatility. This HR volatility threshold is defined as a rolling 10-minute standard deviation of the HR signal of 6 beats per minute. Defining the final sleep window limits as the last, and first high volatility epochs for sleep onset and offset, respectively, is meant to increase the algorithm’s sensitivity at discriminating sedentary time just before or after sleep (e.g., reading in bed) from the sleep window itself.

Finally, the algorithm also labels naps and awakenings, but these were not used in the analysis of the present datasets. Naps are the initial sleep sequences that lie outside a buffer 180-minute window either side of the main sleep window. For awakenings, the algorithm labels all the epochs when the HR rises above a quantile threshold *AV* extracted from the daytime (8am - 10pm) HR ECDF. From these only the sequences longer than 5 minutes are kept and then the sequences separated by less than 5 minutes of sleep are merged and then labeled as the final awakenings.

**Figure 2:**
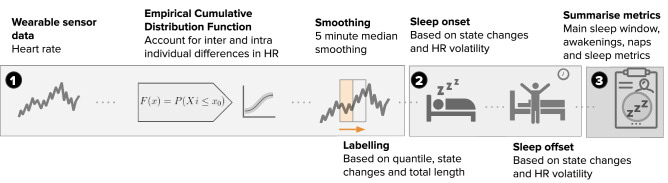
Heart rate sleep algorithm description. The approach can be broken down into three distinct steps. The first step, involves obtaining the wearable sensor HR data, pre-processing that data and setting initial sleep blocks through ECDF quantile thresholds *Q*. Blocks longer than *L* minutes are kept and merged with other blocks if their gap is smaller than *G* minutes. We extract the limits of the resulting blocks as sleep candidate for sleep onset and offset. Next, rolling heart rate volatility is used to refine these candidate times by finding nearby periods where this volatility is high. Finally, nap and awakenings are labeled, the former coming from the candidate sleep blocks not included in the largest sleep window, while the latter are short periods (<60 minutes) within the sleep window when the heart rate exceeds the daytime threshold. A detailed description of this algorithm and parameters used can be found in the methods section. The icons used in this figure are licensed under Creative Commons by thenounproject.com.

**Figure 3:**
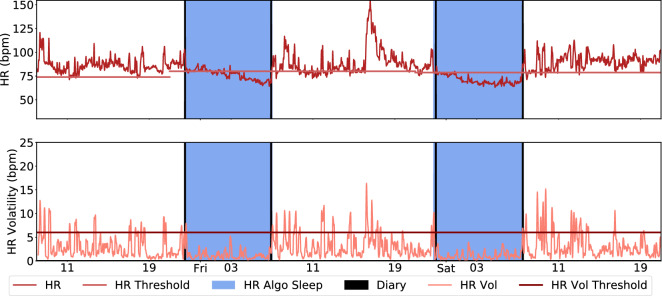
Heart rate sleep algorithm in action for a participant chosen at random. The first step involves setting initial sleep blocks through ECDF quantile thresholds (in this experiment, $Q=.35$). Blocks longer than $L=40$ are kept and merged if the gap between blocks is smaller than $G=60$ minutes. We extract the limits of the resulting blocks as candidate state changes. The bottom panel highlights the use of rolling heart rate volatility to refine these candidate times by finding nearby periods where this volatility is high. The resulting candidate times designate each day’s main sleep window.

Pseudocode for the approach is provided in the Supplementary Material Algorithm 1. A visual overview of the algorithm is provided in Figs. 2 and 3 showcases the application of the algorithm to a random participant trace.

### Evaluation of the proposed approach

We used the four previously described cohorts to evaluate our method against gold-standard measures of sleep using PSG (MESA, Apple Watch PhysioNet) and detailed silver-standard measures through sleep diaries, as opposed to habitual sleep diaries which could be subject to recall bias (BBVS, MMASH). Although an ideal experimental protocol would have multiple days of PSG and free-living wearable sensor data, detailed sleep diaries allowed us to evaluate the algorithm across more than one or two nights, showcasing the strength of our method at discerning both inter- and intra-individual variability.

We performed epoch by epoch evaluation on all four cohorts and derived comparisons regarding the performance of our method with regards to total sleep time (TST), sleep onset and sleep offset time.

#### Evaluation metrics

The following metrics were used to evaluate against the ground truth in each study: differences in onset/offset/total sleep block duration (minutes), mean square error (MSE) and Cohen’s $\kappa$. We evaluated our algorithm systematically for individual HR CDF quantiles $Q \in [0.10, 0.95]$ with step size 0.025, window lengths $L \in [10, 120]$ minutes with step size of 5 minutes, and gap between blocks $G \in [30, 420]$ minutes with step size of 30 minutes, optimizing for MSE.

We defined MSE as:

3 $$\begin{aligned} \text {MSE}_{\text {algo},\text {ground}\_\text {truth}} = \frac{\text{ number } \text{ of } \text{ incorrectly } \text{ labeled } \text{ epochs }}{\text{ number } \text{ of } \text{ epochs }} = \frac{\sum _{i =1}^{n} (\text {algo}_{i} - \text {ground}\_\text {truth}_{i})^2}{n}, \end{aligned}$$

where *algo* and *ground_truth* are the binary labels for an epoch (1 for sleep, 0 for wake) out of *n* epochs in each subject’s time series. Epoch length is specified by the different study cohorts (1 minute in BBVS, 30 seconds in MESA and 15 seconds in both PhysioNet Apple Watch and 5 seconds in MMASH). Thus, if the sleep windows found by the HR algorithm match the ground truth labels exactly, $MSE = 0$. If the algorithm labels all epochs as wake, then MSE is the proportion of sleep in the time series according to ground truth, while if the algorithm and ground truth labels diverge entirely, MSE will be the sum of their sleep proportions out of the total time series. For all four cohorts we performed systematic parameter optimization for best MSE on the basis of quantile, window length and window merge values. We also computed Cohen’s kappa, which is used to determine the classifier agreement with ground truth (PSG or sleep diary), relative to chance^52^. Cohen’s kappa is calculated through $(p_{o} - p_{e})/ (1 - p_{e})$, where p$_{o}$ stands for the percentage of observed classifications with agreement, and p$_{e}$ is the percentage of classifications from hypothetical chance agreement.

The results are represented as the mean ± 95% confidence interval around the mean. The modified Bland-Altman technique was applied to verify the similarities between the different methods. Significant tests were conducted with a two-sided t-test^53^. All statistical analyses were performed with Python 3.8 and SciPy 1.4.1.

We have developed an open-source, python library that provides the code base for our HR method and other well-established techniques for the analysis of sleep and circadian rhythms using accelerometer, actigraphy and heart rate data. The library is called *HypnosPy* and can be found here: https://github.com/HypnosPy/HypnosPy/.

Finally, note that a very detail description of how the evaluation was performed for each one of the datasets is described in the Supplementary Material.

## Results

### Participant characteristics

To develop our heart rate-based sleep inference algorithm, we started our optimal parameter search in a cohort (BBVS) where data was recorded for the entire day. However, benchmarking was inevitable against annotated, but structurally different night-only studies (e.g. MESA) and required separate analyses on whole-day and night-only BBVS datasets selections. The resulting differences in the best parameters stem from the fact that night-time only data rarely comes from free-living conditions and requires separate parameter inference built into our heart-rate algorithm. The optimal parameters from the 24-hour and night-time BBVS analyses were then applied to the other 3 cohorts. Characteristics of cohort, evaluation and validation sets are summarized in Table 2.

**Table 2:** Summary of data set demographics.

Feature	Biobank validation study (BBVS)	Multi-ethnic study of atherosclerosis (MESA)	PhysioNet apple watch	Multilevel monitoring of activity and sleep in healthy people (MMASH)
*Number of participants*	193	2230	31	22
*Age (years, mean(std))*	54.13 (6.95)	68.65 (8.91)	29.42 (8.52)	26.05 (7.12)
*Percent Male*	54.40	46.28	32.26	100
*BMI (mean(std))*	26.21 (3.21)	Not available	Not recorded	23.12 (3.09)

### Evaluation of the algorithm in the BBVS

The results of the hyper-parameter search on the BBVS dataset are summarized in Table 3 and histograms detailing the results are shown in Figs. S2 and S3. While the main application of our algorithm would be on full-day data, we also experiment with night-only datasets. The hyper-parameters picked by the grid-search method are used in all the other experiments we report and shows the generalizability of our approach.

The set of hyper-parameters varies depending on the use case. For the whole day experiment, the optimal HR quantile threshold was 0.325, in line with the fact that we usually spend about one-third of our days sleeping. For the night-only experiment, both the HR quantile and the gap-merging threshold are much higher, indicating that the best results occurred when the algorithm prioritized a single sleep block and aimed to ignore activity outside this block.

**Table 3:** Optimal hyper parameters extracted from a grid search on the BBVS dataset for both full-day and night-only data. These parameters are used accordingly on the other three datasets studied in this work.

Scenario	HR quantile threshold (Q)	Minimum length (L)	Gap merging threshold (G)
Full Day	0.325	20	90
Night Only	0.800	20	420

The results of the evaluation on the BBVS study are summarized in Table 4. Our HR algorithm optimized on the full day yielded an MSE of 0.06 and estimated a TST on average 9.84 minutes longer compared to sleep diaries. We compared this result with the angular change approach shown in Table 5. The best performing wrist-worn device (non-dominant wrist) had an overestimation of 192 minutes compared to the sleep diaries. The results across all three accelerometers for this approach were comparable as summarized in Table 5, each yielding an MSE of 0.17.

**Table 4:** Results of applying the HR algorithm on the BBVS dataset for both full-day and night-only data. Comparisons are made against sleep diaries. BBVS TST for diaries mean ± 95% CI = 7.739 ± 0.073 hours (464.34 ± 4.38 minutes).

Sleep parameter	Metric	HR algorithm (Full day)	HR algorithm (Night only)	*p*-value
		(mean ± 95% CI)	Value (mean ± 95% CI)	
Total sleep time	Time difference (minutes)	−2.70 ± 5.74	12.80 ± 3.89	< 0.00
	MSE	0.06 ± 0.00	0.04 ± 0.00	< 0.00
	Cohen’s kappa	0.86 ± 0.00	0.90 ± 0.00	< 0.00
Sleep onset	Time difference (minutes)	−0.49 ± 5.67	−4.59 ± 3.27	0.158
Sleep offset (Wake Up)	Time difference (minutes)	−3.19 ± 4.80	8.20 ± 2.88	< 0.00

**Figure 4:**
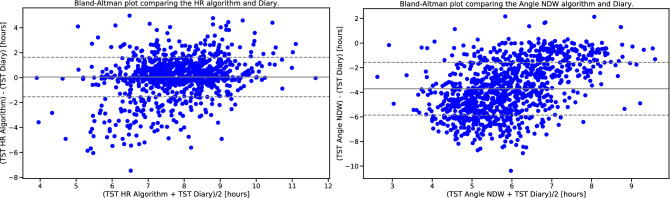
Modified Bland-Altman plot for BBVS. Modified Bland-Altman plot on the left shows the TST differences (delta) between the full-day HR algorithm and diary in the Y-axis and the X-axis shows the TST average for every participant. The figure to the right shows the same comparison for the angle algorithm and diaries in BBVS. Dashed lines represent limits of agreement (LoA) which are defined as the mean difference ± 1.96 SD of differences. TST: total sleep time.

**Figure 5:**
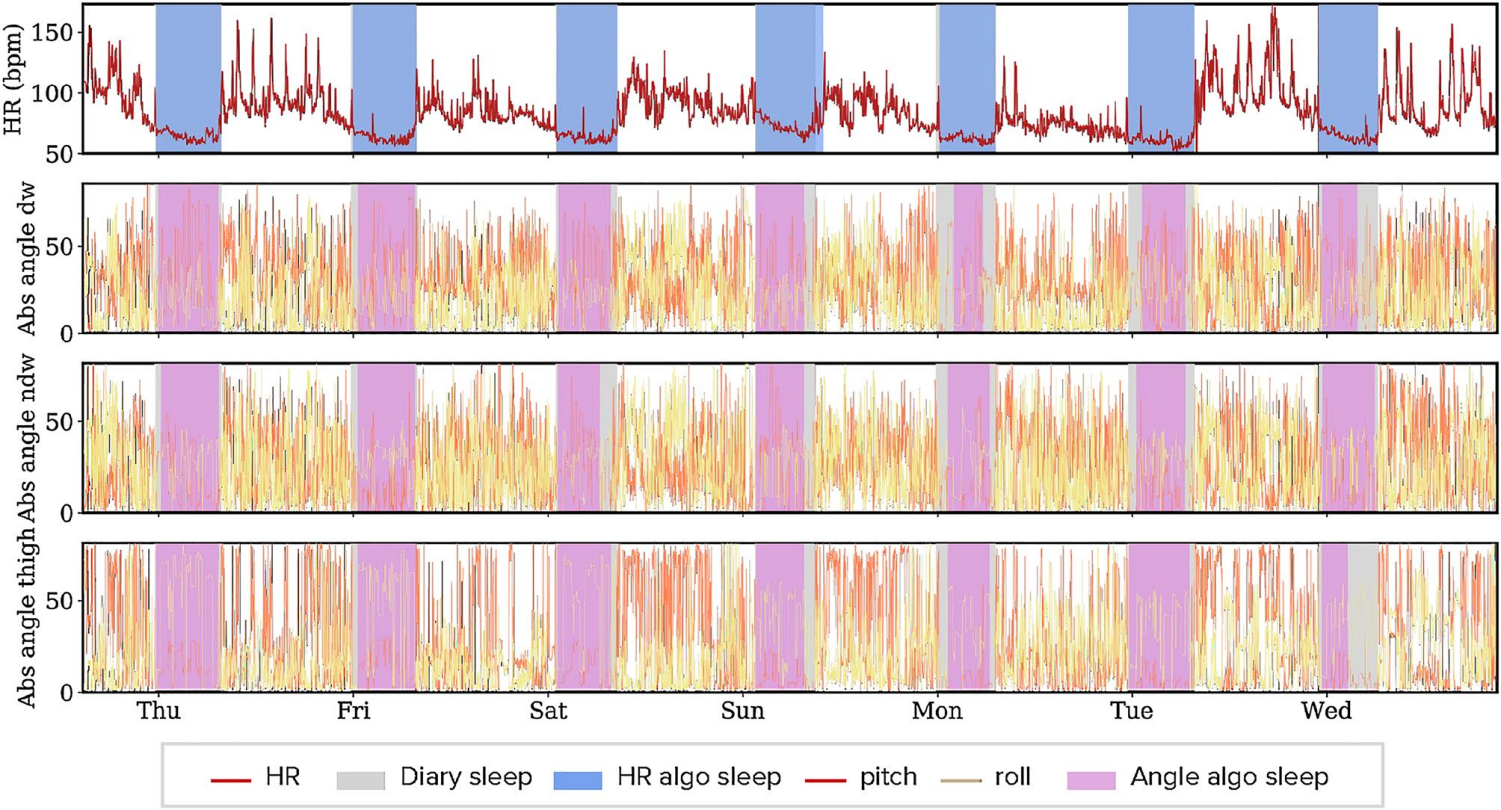
Example participant (chosen at random), showcasing estimated sleep through the heart rate sleep window algorithm, sleep diary sleep onset and offset and angle changes for both wrists and the thigh accelerometers. The algorithm picks up subtle sleep regularity differences at a participant level. This approach overlaps more closely to the sleep diary than any of the accelerometer-based approaches. Notice that, for the angle change approach, the algorithm is more effective on the non-dominant wrist accelerometer than on the dominant wrist or thigh accelerometer for most nights. TST: total sleep time.

Our HR model estimated sleep onset on average 8.84 minutes later than sleep diary while the angular change approach on the non-dominant wrist resulted in an average overestimation of 87.88 minutes. For diary-based sleep offset, our HR algorithm the estimation was on average 1.00 minute earlier, while for the angular change approach that estimation was 104.33 minutes earlier for the non-dominant wrist. Modified Bland-Altman plots for the HR and angle approaches against sleep diary for the BBVS cohort are presented in Fig. 4. Finally, Fig. 5 showcases an example BBVS participant analysed with both the HR and angle change sleep algorithms.

**Table 5:** Comparison of angle algorithm performance for the BBVS dataset by the limb on which the device was worn. All participants wore devices on their dominant (dw) and non-dominant (ndw) wrist as well as on their thigh. The best performance metrics were obtained for the non-dominant wrist device, but thigh wearables gave the least time differences overall in terms of total sleep time (TST), sleep onset and offset. BBVS TST for diaries mean ± 95% CI = 7.739 ± 0.073 hours (464.34 ± 4.38 minutes).

Sleep parameter	Metric	Angle change algo. (ndw)	Angle change algo.(dw)	*p*-value	Angle change algo. (Thigh)	*p*-value
		Value (mean ± 95% CI)	Value (mean ± 95% CI)	ndw-dw	Value (mean ± 95% CI)	ndw-thigh
Total sleep time	Time difference (min.)	222.64 ± 7.78	218.96 ± 7.92	0.271	214.90 ±8.08	0.048
	MSE	0.16 ± 0.00	0.16 ± 0.00	0.052	0.16 ± 0.00	0.291
	Cohen’s kappa	0.58 ± 0.01	0.59 ± 0.01	0.027	0.58 ± 0.01	0.650
Sleep onset	Time difference (min.)	−100.99 ± 6.63	−96.00 ± 7.01	0.167	−96.07 ± 7.85	0.250
Sleep offset (Wake Up)	Time difference (min.)	121.65 ± 7.05	122.96 ± 7.43	0.727	118.82 ± 8.11	0.501

### Evaluation and fine-tuning of the algorithm in the MESA study

**Figure 6:**
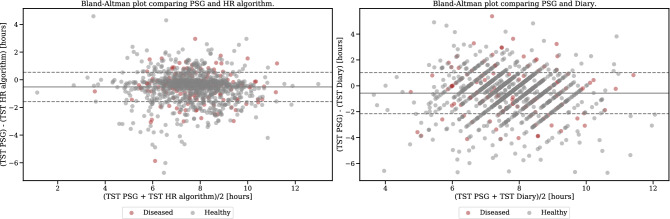
Modified Bland-Altman plot for MESA. Modified Bland-Altman plot on the left shows the TST differences (delta) between the HR algorithm and PSG in the Y-axis and the X-axis shows the TST average for every participant. The figure to the right shows the same comparison for the sleep diaries and PSG in MESA. Further, healthy participants are color coded in blue for both plots and participants that were diagnosed with sleep disorders are shown in orange.

To validate the approach used on the BBVS cohort, we used the MESA dataset, which contains both sleep diary and PSG data. In addition, some MESA participants had been formally diagnosed with sleep disorders. The results are detailed in Table 6. Our HR-based algorithm and annotations from sleep diaries and PSG were tested against one another, resulting in the modified Bland-Altman plots in Fig. 6.

**Table 6:** Results for the MESA dataset. Both the HR algorithm and sleep diaries are evaluated against PSG. Results are also shown for the subset of healthy participants and participants with sleep disorders. MESA TST for PSG mean ± 95% CI = 7.433 ± 0.079 hours (445.95 ± 4.71 minutes). N=1,154.

Sleep parameter	Metric	HR algorithm	Sleep diary	*p*-value
		Value (mean ± 95% CI)	Value (mean ± 95% CI)	
Total sleep time	Time difference (min.)	−55.04 ± 3.75	−34.04 ± 5.50	< 0.00
	MSE	0.11 ± 0.01	0.13 ± 0.01	< 0.00
	Cohen’s kappa	0.59 ± 0.02	0.62 ± 0.01	0.01
Sleep onset	Time difference (min.)	39.72± 3.01	6.25 ± 3.30	< 0.00
Sleep offset (Wake Up)	Time difference (min.)	−15.32 ± 2.52	−27.79 ± 4.86	< 0.00

Healthy participants (N = 965)				
Total sleep time	Time difference (min.)	−56.12 ± 4.32	−36.05 ± 6.05	< 0.00
	MSE	0.11 ± 0.01	0.13 ± 0.01	< 0.00
	Cohen’s kappa	0.59 ± 0.02	0.62 ± 0.02	0.013
Sleep onset	Time difference (min.)	40.36 ± 3.30	6.12 ± 3.64	< 0.00
Sleep offset (wake up)	Time difference (min.)	−15.76 ± 2.78	−29.93 ± 5.34	< 0.00

Participants with sleep disorders (N = 189)				
Total sleep time	Time difference (min.)	-49.51 ± 9.47	−23.75 ± 13.05	< 0.00
	MSE	0.11 ± 0.01	0.13 ± 0.01	0.071
	Cohen’s kappa	0.58 ± 0.04	0.60 ± 0.04	0.448
Sleep onset	Time difference (min.)	36.45 ± 7.41	6.92 ± 7.82	< 0.00
Sleep offset (wake up)	Time difference (min.)	−13.07 ± 5.96	−16.84 ± 11.66	0.540

Results from the MESA cohort confirmed that the HR-based algorithm was non-inferior to human-annotated sleep. For both healthy sleepers and participants with sleep disorders, the average MSE for the PSG sleep-wake labels was 0.11 (versus 0.13 for sleep diaries). The superior performance of using HR was also reflected in a better Cohen’s kappa for all three analyses. Both approaches underestimated the TST compared to PSG-derived labels by -55.04 and -34.04 minutes, respectively for the HR algorithm and sleep diaries, a difference that was statistically significant. Interestingly, our algorithm was better at inferring sleep offset (-15 minutes compared to PSG labels, versus about -30 minutes for sleep diaries), but worse at detecting sleep onset (40 minutes, versus 6 minutes for the sleep diary). As the MESA study only recorded night-time data, the HR quantile optimal for the 24-hour BBVS data was not suitable. Instead, we benchmarked the MESA data against the best HR quantile for a night-only window of the BBVS cohort, which was 0.80. This led to an MSE of 0.11 between the HR algorithm and PSG sleep labels.

### Validation of the algorithm in the PhysioNet apple watch polysomnography study

Our algorithm was applied to data obtained from a commercial wrist-worn wearable and evaluated against gold-standard PSG-labelled sleep. Given the presence of triaxial accelerometry, we could compare the HR- and angle change-based methods. Using the optimal parameters derived from the night-only BBVS data, the HR algorithm resulted in an MSE of 0.07, while the wrist-based angular change approach yielded an MSE of 0.12. TST deviation was -29.07 minutes for the HR approach and 44.39 for the angle change approach. Sleep onset time deviation was 20.73 minutes for the HR approach and -21.77 for the angle change approach, while the difference was -8.34 and 22.61 for sleep offset. However, Cohen’s kappa was slightly lower for the HR approach (0.59) than for the angle change algorithm (0.71). These results are summarized in Table 7.

**Table 7:** Results for the PhysioNet Apple Watch dataset. The table presents results for both the HR and angle change algorithm for total sleep time, sleep onset and sleep offset in the PhysioNet Apple Watch dataset. PhysioNet Apple Watch TST for PSG mean ± 95% CI = 7.165 ± 0.544 (429.89 ± 32.65 minutes). ndw: Non-dominant Wrist. N = 22.

Sleep parameter	Metric	HR Algorithm	Angle change algorithm (ndw)	*p*-value
		Value (mean ± 95% CI)	Value (mean ± 95% CI)	(n = 22)
Total sleep time	Time difference (minutes)	−29.07 ± 13.38	44.39 ± 40.01	0.001
	MSE	0.07 ± 0.03	0.12 ± 0.08	0.277
	Cohen’s kappa	0.59 ± 0.12	0.71 ± 0.13	0.234
Sleep onset	Time difference (minutes)	20.73 ± 5.45	−21.77 ± 29.77	0.008
Sleep offset (Wake Up)	Time difference (minutes)	−8.34 ± 11.98	22.61 ± 31.01	0.056

### Validation of the algorithm in the MMASH study

Our final set of evaluations took place in the MMASH cohort, which included both HR and triaxial accelerometer data recorded continuously for full-day periods. We evaluated HR- and angle change-based algorithms against detailed sleep diaries. Starting from the best parameters from full day BBVS data, the HR method gave an MSE of 0.11 and TST difference of 17.64 minutes, with a Cohen’s kappa of 0.75. As additional validation, we repeated analysis on MMASH data from the night period only, using the night-only BBVS best parameters, with a similar MSE result of 0.09 against sleep diaries. On the other hand, the angle change approach resulted in an MSE of 0.10 and Cohen’s kappa of 0.78, but the TST deviation was substantially worse, yielding a total sleep time difference of -55.86 minutes. Full results for the MMASH cohort are presented in Table 8. Similar to BBVS, results of the optimal parameter search for the MMASH cohort can be found in the Supplementary Material Fig. S4.

**Table 8:** Results for the MMASH dataset. The table presents results for both versions of the HR algorithm and compares them to the angle change algorithm for total sleep time, sleep onset and sleep offset in the MMASH dataset. MMASH TST for diaries mean ± 95% CI = 6.200 ± 0.622 hours (371.98 ± 37.33 minutes). ndw: Non-dominant Wrist. N = 21.

Sleep param.	Metric	HR Algo. Full Day - HRD	Angle change Algo. (ndw)	*p*-value	HR Algo. Only Night - HRN	*p*-value
		Value (mean ± 95% CI)	Value (mean ± 95% CI)	(ndw - HRD)	Value (mean ± 95% CI)	(ndw - HRN)
Total sleep time	Time difference (min)	17.64 ± 47.78	−55.86 ± 42.67	0.009	−34.36 ± 35.24	0.366
	MSE	0.11± 0.04	0.10 ± 0.04	0.487	0.09 ± 0.03	0.742
	Cohen’s kappa	0.75 ± 0.10	0.78 ± 0.09	0.465	0.80 ± 0.06	0.692
Sleep onset	Time difference (min)	−39.14 ± 44.60	9.55 ± 34.87	0.127	36.00 ± 24.85	0.204
Sleep offset	Time difference (min)	−21.50 ± 33.85	−46.31 ± 31.64	0.080	1.64 ± 33.09	< 0.00

## Discussion

Objective and unobtrusive measurement of sleep in large, free-living populations at scale will help facilitate epidemiological investigations powered to explore the relationships between sleep, behavior and disease. This is helped by the rapid adoption of wearables. However, most commercial devices use proprietary algorithms or do not thoroughly validate against gold-standard measures. Similarly, conventional algorithms often rely on device specific metrics, such as counts, requiring extensive adaptation for each device and cohort tested, as well as a predefined search window through expert annotations or sleep diaries. This often renders evaluation across devices and without sleep diaries impractical.

We presented a device-agnostic algorithm that exploits the HR-sensing capabilities present in most modern wearable devices. The proposed method relies on the established changes in HR that occur when individuals transition from wake to sleep^54^. Hence, our approach is versatile over individual fitness levels or illness and could be used amongst those who sleep outside the night period (as explored in Fig. 3). These qualities may be particularly relevant when evaluating sleep in populations of shift workers, in countries where sleep timing changes due to seasonality or where cross-cultural sleep differences are observed^55^. The algorithm is also in theory capable of inferring naps and fragmented sleep episodes. Naps are as default defined as sleep periods shorter than one hour more than 3 hours apart from the main sleep window. Polyphasic sleep is accounted for if more than 2 sleep episodes exceed 90 minutes in length. Given the nature of the datasets used for this study, we could not systematically evaluate the performance of the method in naps, which is something we aim to do in future studies. This could improve the performance of the algorithm at detecting wake periods that occur during or close to sleep and allow us to include additional sleep metrics such as wake after sleep onset (WASO) and number of awakenings during the sleep period, which were not evaluated in this paper. This approach could help address and overcome the long-standing issue of low specificity for actigraphy-based sleep scoring when evaluated against PSG and complement studies that have attempted to achieve this by incorporating other types of wearable and environmental sensors.

### Performance evaluation and benchmarking against state-of-the-art methods

We validated our HR-based algorithm using four cohorts: BBVS, MMASH, PhysioNet and MESA. Both BBVS and MMASH include free-living HR, movement and sleep diary data for multiple days. By contrast, PhysioNet and MESA provide lab-based HR data and gold-standard PSG. Our aim was to optimize the algorithm in the BBVS free-living dataset and then validate it against gold-standard measures in the other three cohorts. Through this process, we identified the range of parameters (*Q*, *L*, *G*) that produce the best results in free-living conditions, allowing for application and deployment in the absence of any ground truth.

For the first evaluation in the BBVS study, the method we propose performed strongly in free-living conditions, with an average time deviation for TST compared to non-habitual sleep diaries of 2.70 minutes. The optimal parameter search used both full-day and night-only HR to analyze how the availability of sensor data or the design of the experiment affect the best parameter choice. The parameter search for the optimal MSE was performed based on quantile, window merge and window length values and is presented in Supplementary Fig. S2. The optimal full-day parameters for this cohort were 0.325 for quantile (*Q*) and 20 minutes for window length (*L*) with a time merge block of 90 minutes (*G*). This resulting optimal quantile makes intuitive sense as it represents about 8 hours, approximately the expected time spent sleeping for most individuals in a day. For the night-only data, the best parameters were 0.80 for the quantile, 20 minutes for the window length and a time merge block of 420. The higher quantile for the night period also makes sense as a lower percentage of the total time would have been spent in active behaviour and participants would have been more likely to be sedentary and supine later in the day. The MSEs against sleep diaries were comparable for night-only and full-day data (0.04 vs. 0.06), which shows that a window-agnostic analysis does not lead to a significant loss of performance. This flexibility allows discovery of non-standard sleep patterns, such as biphasic sleep or daytime sleep in shift workers.

The algorithm detected self-reported sleep offset (wake up) better than sleep onset, yielding a time difference between 1.64 (MMASH) and -15.32 (MESA) and between -0.49 (BBVS) and 39.72 (MESA) minutes, respectively. These results may be affected by two factors. First, sleep diaries recording onset and offset are self-reported and may be inaccurate. While sleep offset is relatively straightforward to annotate as most people wake up with alarm clocks, the exact time of sleep onset cannot be recorded, and is prone to measurement bias, if attempted at the time, or recall bias, if filled in the next day. The quality of self-reported sleep may vary based on the sleep onset latency of each participant for each night. Second, the considerable differences in the MESA dataset are likely due to the experiments starting when participants were already supine in bed, yielding limited variance on the HR signal as opposed to other full-day datasets. Nevertheless, the method’s performance across a diverse population and multiple nights of recording showcases its potential for free-living applications. Supplementary Table S2 also evaluates the performance of the method against traditional actigraphy based methods, showcasing that these traditional approaches were not meant to be deployed in full-day recordings and greatly benefit from the inclusion of a sleeping window for guidance. This sleeping window has traditionally come from sleep diaries and can now be derived using the proposed HR method in the absence of these diaries.

Finally, in the BBVS cohort, we evaluated the performance of an angle change-based algorithm inspired by previous work^29,56^ leveraging the multiple accelerometers available to evaluate angle-based postural changes. We found this approach to be valuable, but the results were more modest than those of our method, yielding a total sleep time MSE of 0.16 and a time deviation of 222.64 minutes for the non-dominant wrist device. Using the combined pitch and roll approach versus only the z-angle did not significantly alter the results. These results suggest that when HR is available, it should be used preferentially, but when it is missing, triaxial accelerometry is a valuable secondary option.

### Full-day versus night-only evaluation

The algorithm was also evaluated in the MESA cohort, a large, diverse population where PSG was available, alongside self-reported sleep diaries. We started from the BBVS night-only optimized parameters, with additional segmentation into healthy and sleep disorder-diagnosed population subsets. This HR algorithm analysis yielded the results reported in Table 6. In MESA, the TST deviation versus PSG measures of sleep was -55.04 minutes and MSE of 0.11 for the full population, whereas the same comparison between PSG and sleep diaries yielded a total sleep time deviation of -34.04 minutes and MSE of 0.13. This shows that our HR-based method can reliably and objectively monitor sleep in the absence of PSG and performs better than sleep diaries.

It is also worth noting that the HR approach was better at detecting PSG measured sleep offset (wake up) with a time difference versus PSG of -15.32 minutes compared to the -27.79 for the diaries. Furthermore, comparable results were obtained on the separate healthy and sleep disordered subgroups, suggesting our method may be valuable when trying to diagnose these sleep disorders or monitor the sleep of those already diagnosed. To the best of our knowledge, this is the first study that conducts these types of sensitivity analyses on a subset of sleep disorder subjects to show the validity of the proposed method in these individuals. Future work should benefit from a larger sleep disorder population sample with free-living data. Our method could offer additional insights through its ability to detect naps and daytime sleep.

We then examined the performance of the HR algorithm in the PhysioNet cohort that recorded concurrently Apple Watch data and a night of PSG. This experiment followed a similar protocol to that of the MESA study, including the use of the BBVS night-only optimal parameter benchmark. In this cohort, the HR algorithm yielded an MSE of 0.07 and a time-deviation of -29.07 minutes when compared to gold-standard measures of PSG sleep. These results showcase the photoplethysmography-derived HR’s potential for sleep inference in commercial-grade wearables. Our approach should transfer well to other similar devices. These have been shown to be reliable at detecting resting and sleeping heart rate, which are critical for our method, despite being less reliable at higher heart rates due to activity-associated artifacts^57^. We also examined the angle change approach in this cohort, but this method performed less well than it did in BBVS, yielding an MSE of 0.12 and a TST deviation of 44.39 minutes.

Finally, we tested our method in the MMASH cohort, where free-living HR, movement and sleep diary data was available for whole days. As additional validation, we split this data into full-day and night-only subsets to replicate the BBVS analysis. Using BBVS-optimised parameters for these subsets, the MSEs against sleep diaries were 0.11 for the full-day and 0.09 for the night-only analyses. For the full-day data, the TST deviation was 17.64 minutes. As a comparison, the angle change approach resulted in an MSE of 0.10 and total time deviation of -55.86.

The results show the HR algorithm to be versatile across datasets. Pre-derived optimal parameters can be run quickly on other cohorts, with the caveat of choosing appropriate full-day and night-only values, as a sleep time prior is built into the *Q* parameter. If the lowest possible MSE value is desired, then the analysis can be further optimized with a cohort-specific parameter search.

### Further implications and future work

One important limitation of the BBVS and MMASH studies is that they did not include PSG-derived ground truth sleep annotations. An ideal experimental protocol would have multiple days of PSG and free-living wearable sensor data, but detailed sleep diaries allowed us to evaluate the algorithm across more than one or two nights. This showed the usefulness of our method when both inter- and intra-individual variability are important. Similarly, the accelerometers included in these studies offer an important perspective on how methods based on postural angle changes fare against or add to our approach. In ideal circumstances, full-day HR would have been available in both the MESA and PhysioNet cohorts. Exposure of the HR algorithm to non-sedentary wake behaviour would have further optimized it. However, the results in these two datasets showcase the validity of our approach even under constrained laboratory conditions.

While in this work we focused on sleep onset and offset evaluation, another limitation of this paper is the lack of an evaluation of other important sleep quality metrics, such as WASO and number of awakenings during the sleep period. In future work, we will also aim to study these metrics and how to integrate our proposed HR algorithm with traditional sleep-wake algorithms (i.e.: Scripps-Clinic^22^ or Oakley^58^) to improve their sensibility to detect wake during sleep.

Future studies should explore the robustness of the HR-based algorithm in special cohorts such as inpatients. As the algorithm relies on HR signals already monitored continuously for other medical purposes, no additional accelerometer sensor would be required. Accurately labelling sleep in inpatients is challenging due to other factors that influence HR range and variability, such as limited mobility, fever, medication, physiological and psychological stress, drug and alcohol use and cardiovascular conditions. However, objectively monitoring sleep without additional obtrusion could help improve sleep quality during hospital stays, which is a challenge for most patients^59^, and hence promote both healing and patient satisfaction. Moreover, optimization of the angle change approach should be explored independently of HR and beyond those reported in van Hees et al.^29^, which we used directly. Parameter optimization could yield stronger, more generalizable outcomes for this approach. Finally, our method could be used in tandem with other established activity-based approaches where multimodal settings are available. For instance, using conditional programming, traditional methods could complement our HR algorithm to detect awakenings and assist in deriving conventional and novel sleep metrics.

## Conclusion

Overall, our work highlights the potential of HR to detect the sleeping window not only in research and clinical contexts, but also in ecologically-valid free-living conditions. This frees sleep monitoring from the constraints of PSG and diaries without compromising its objectivity. The low effort involved in collecting and analysing inferred sleep data through our method, coupled with fewer exclusions due to technical errors, incomplete diaries or dropout would likely result in larger and more diverse study cohorts that can be monitored over longer times. For instance, few studies have the means to adequately test the longitudinal and synergistic association between sleep quality and disease. Where this has been attempted, sleep data is often collected through questionnaires^60^ or after short, arbitrary follow-up periods. These studies could have missed trends that significantly influence health status over months or years.

To conclude, our proposed method was shown to be a valuable device-agnostic tool that can infer sleep in both free-living and laboratory conditions without the need for diaries. As highlighted by Depner and colleagues^61^, our evaluation could enable the translation of findings from laboratory sleep studies to large-scale cohort studies and clinical trials, by providing objective and annotation-free sleep inference valid across multiple wearable devices and recorded time windows.

## Supplementary Information

Supplementary Information.
