# Supplementary material for: Detecting sleep outside the clinic using wearable heart rate devices
Source: Sci Rep. 2022 May 13;12:7956. doi: 10.1038/s41598-022-11792-7 (PMC9106748; doi:10.1038/s41598-022-11792-7)
Supplement: Supplementary file 1 — Supplementary Information. [file 41598_2022_11792_MOESM1_ESM.pdf]

Appendix

Detecting sleep outside the clinic using wearable heart rate devices

Ignacio Perez-Pozuelo, Marius Posa, Dimitris Spathis, Kate Westgate, Nicholas Wareham, Cecilia Mascolo, Søren Brage, and Joao Palotti

In Review – Scientific Reports

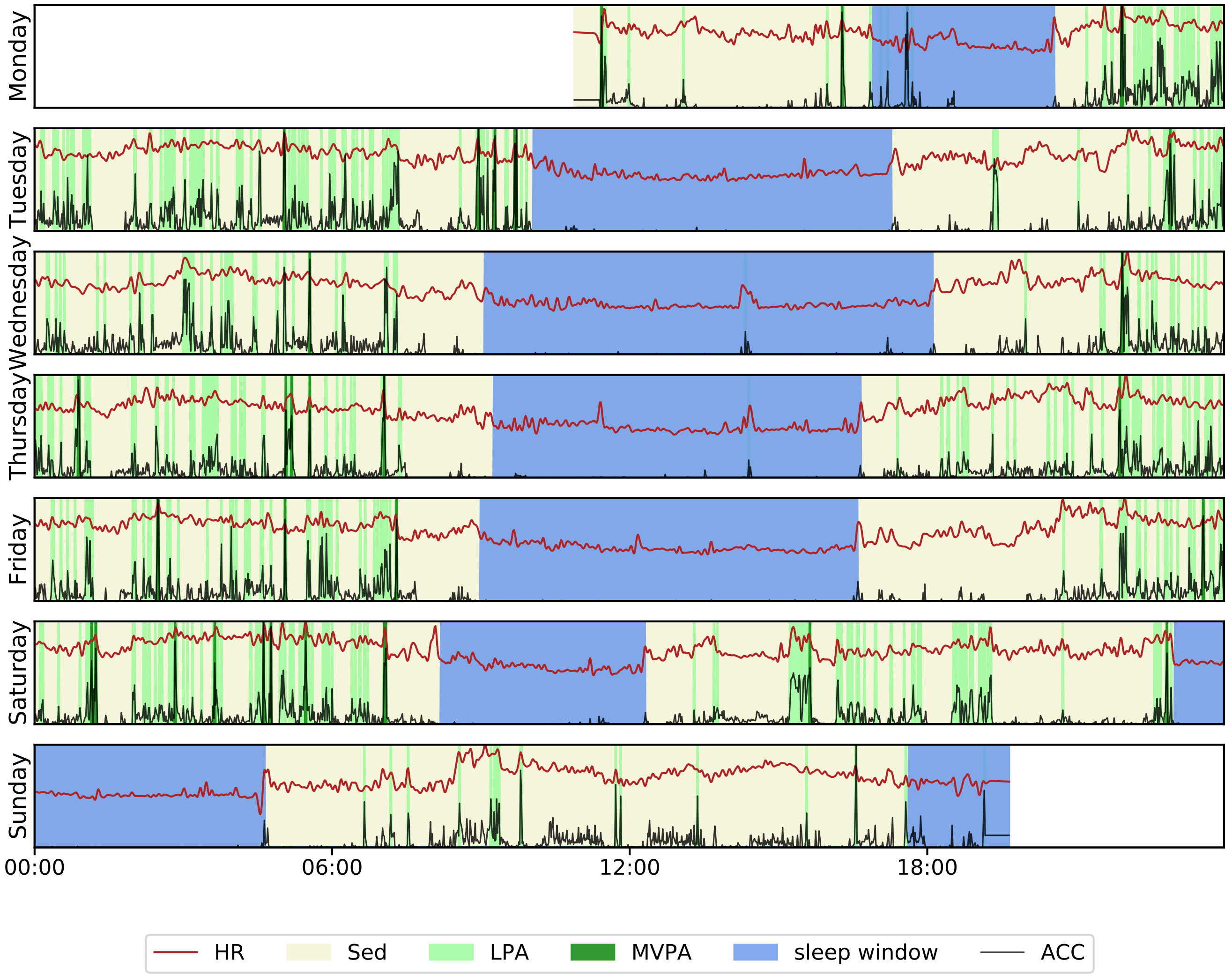

Figure S1. Applying the HR sleep algorithm on a shift worker. The free-living trace shows the subtle changes for day of the week picked up by the algorithm (inference showcased as sleep window, in blue), with 2 sleep windows detected on Saturday, when they were not at work during the night. HR: Heart Rate; Sed: Sedentary; LPA: Light Physical Activity; ACC: Acceleration.

Details on the Data Sources

The UK Biobank Validation Study (BBVS)

Participants of the BBVS study were recruited from the Fenland study[4]. In brief, 193 participants were recruited between the ages of 40 and 70, with a BMI between 20 and 50kg $\cdot$ m^{-2}. Recruitment aimed to balance age, sex, and BMI distributions. Participants were invited to attend an assessment centre on two separate occasions, separated by a free-living period of 9 to 14 days during which they wore three waveform triaxial accelerometers (dominant and non-dominant wrists and thigh) as well as a combined movement and heart rate sensor. During the free-living period, participants were asked to keep a detailed log of their sleep, by recording the time they fell asleep and woke up on a daily basis. Ethical approval for the study was obtained from Cambridge University Human Biology Research Ethics Committee (Ref: HBREC/2015.16). All participants provided written

Table S1. Prevalence of sleep disorders in the subset of MESA dataset used in this work (n=1,154) against the reported prevalence in the general population.

Sleep Disease	Participants in Mesa (%)	Prevalence in the population
Restless Legs Syndrome	59 (5.11%)	1.9 - 4.6%[1]
Insomnia	67 (5.81%)	6%[2]
Sleep Apnea	95 (8.23%)	3-7%[3]
Any of the above	189 (16.38%)	-

informed consent. Full details of the BBVS study are described elsewhere[5].

Participants were fitted with a combined heart rate and movement sensor (Actiheart, CamNtech, Cambridgeshire, UK), measuring heart rate and uniaxial acceleration of the trunk every 15 seconds[6]. In addition, participants were fitted with three waterproof triaxial accelerometers (AX3, Axivity, Newcastle upon Tyne, UK); one device was attached to each wrist with a standard wristband, and one to the anterior midline of the right thigh using a medical-grade adhesive dressing. These devices were set up to record raw, triaxial acceleration at 100Hz with a dynamic range of $\pm 8g$. BBVS participants were asked to wear all four devices continuously for the following 8 days and nights while continuing with their usual activities. In addition, they were asked to complete a diary of their sleep onset and wake times daily. This ensured that any small changes in onset and offset of sleep were captured during the recording period.

Following the download of the devices, the combined sensor heart rate data was cleaned and non-wear periods identified by the combination of non-physiological heart rate and prolonged periods of no movement[7]. All signals from the triaxial accelerometers were re-sampled to a uniform 100Hz signal by linear interpolation, and then calibrated to local gravity using a well-established technique[8,9]. Periods of non-wear were classified on the basis of windows comprising an hour or more wherein the device was inferred to be completely stationary, where stationary is defined as standard deviation in each axis not exceeding the approximate baseline noise of the device itself ($13 \cdot$milli-g). All non-wear periods were removed from the analysis. Additionally, pitch, roll and z-angles for all three accelerometry devices were calculated enabling angular postural assessments and direct comparisons to previously established approaches which only rely on acceleration data[10,11]. The residual acceleration signal can be interpreted as a measurement of the rotated gravitational field vector which can then be used to determine the accelerometer's orientation angles (the conventional pitch and roll and z-angle, defined as the dorsal-ventral direction[10,11]). Angles for each device were derived according to these formulae:

$$Pitch = \frac{tan^{-1}\left(\frac{Y}{\sqrt{X^2+Z^2}}\right) * 180}{\pi} \tag{1}$$

$$Roll = \frac{tan^{-1}\left(\frac{X}{\sqrt{Y^2+Z^2}}\right) * 180}{\pi} \tag{2}$$

$$Z\text{-}angle = \frac{tan^{-1}\left(\frac{Z}{\sqrt{X^2+Y^2}}\right) * 180}{\pi} \tag{3}$$

The accelerometry and heart rate signals were summarized to a common time resolution of one observation per 30 seconds and the time-series were aligned. Participants were excluded from the final analysis if they had less than 72 hours of concurrent wear data (three full days of recording from all four devices). Participants with less than 3 nights of concurrent wear and diary data were excluded from the final analysis. After these pre-processing steps, the resulting analytical sample was of 158 participants. Three of these participants were on cardioreactive medication and two were taking betablockers.

Multi-Ethnic Study of Atherosclerosis (MESA)

The Multi-Ethnic Study of Atherosclerosis (MESA) is a multi-site prospective study that includes 6,814 men and women who identify as White, Black/African American, Hispanic, or Chinese, and are between the ages of 45-84[12,13]. Participants in this study were followed prospectively to evaluate risk factors for cardiovascular disease. 2,237 MESA participants are enrolled

in a sleep exam (MESA Sleep Ancillary Study[14]), which includes seven days of wrist-worn actigraphy, one full overnight unattended polysomnography (wrist-worn actigraphy collected concurrently), and a sleep questionnaire. MESA participants who reported regular nighttime use of nocturnal oxygen or positive airway pressure devices were excluded from participation.

The MESA Sleep Study was conducted using a Compumedics Somte System for PSG, which includes the ECG signals here used to derive HR and HRV and their associated features, alongside an Actiwatch Spectrum from Philips Respironics (Pennsylvania, USA) to record actigraphy data. This device captures measurements of movement defined as "activity counts" (`https://www.salusa.se/Filer/Produktinfo/Aktivitet/TheActiwatchUserManualV7.2.pdf`) and aggregates them into 30 second epochs. The Actiwatch was securely fastened to participant's non-dominant wrist. These actigraphy signals and their associated features can be derived in most research-grade wearable devices. The sensors for the Compumedics PSG comprised: cortical EEG, bilateral EOG, chin EMG, abdominal and thoracic respiratory inductance plethysmography, airflow, ECG, leg movement sensor and finger pulse oximetry. These sensors collected three types of signals: bioelectrical potentials (EEG, EOG, EMG, ECG), waveforms received from transducers (thermistors on the airflow devices, inductance respiratory bands, piezo leg sensors and position sensors from the leg device) and auxiliary devices (oximetry measures of oxyhemoglobin saturation and nasal pressure records). Full details of the setup, protocol and sampling rates are available elsewhere (`https://sleepdata.org/datasets/mesa/pages/equipment/montage-and-sampling-rate-information.md` and `https://sleepdata.org/datasets/mesa/files/documentation`). All participants included in our study had at least one full night of PSG recording with concurrent actigraphy and ECG. All nocturnal recordings were transmitted to a centralized reading center at the Brigham and Women's Hospital (Boston, MA, USA) and data was scored by trained technicians using AASM guidelines.

For this study, we synchronized PSG, ECG and actigraphy records into 30-second sleep epochs for a subset of 1,743 out of the 2,237 participants included in the original study. A total of 494 participants were excluded on the basis of: (1) lack of concurrent PSG, ECG and actigraphy data; (2) lack of sufficient quality standard data ($<$3h of usable data from the concurrent three sensing methods); or (3) lack of data integrity or misalignment of data, removing the resulting actigraphy outlier epochs based on human expert annotations. These outliers resulted from either non-wearing periods or equipment failure periods. For actigraphy epochs labeled as outliers, their corresponding HR/HRV epochs were also removed[15]. Further, given that some participants records comprised almost no PSG-labelled wake, which is unrealistic for free-living recordings and far removed from the general 24-hour HR quantile assumption of the algorithm, we only included participants who had at least 30 minutes of wake time prior to sleep onset and a maximum of 240 minutes after sleep offset, resulting in a total of 1,154 participants.

To obtain HR information, we used the QRS complexes (R-points) detected using Compumedics Somte (Abbotsford, VIC, Australia) software Version 2.10 (Builds 99 to 101). The R-points were classified as normal sinus, supraventricular premature complex or ventricular premature complex. For the data cleaning, filtering and noise removal, we used the Python package HRV-analysis (`https://pypi.org/project/hrv-analysis/`). First, RR interval outlier data was filtered using a threshold method, with a range between 300 to 2000 ms, based on the approach previously described by Tanaka et al.[16], then ectopic beats were removed by through the methods described in Malik et al.[17]. After this step was completed, we linearly interpolated the removed R-points and we grouped the RR intervals into 30 seconds epochs.

All data used from the MESA Sleep Ancillary study used in this work is publicly available from the National Sleep Research Resource repository (`https://sleepdata.org/datasets/mesa`). Institutional Review Board approval was obtained at each MESA study site (Wake Forest University School of Medicine, Northwestern University, University of Minnesota, Columbia University, University of California Los Angeles and the Johns Hopkins University). All participants provided written informed consent.

A number of common sleep disorders were identified and logged for the MESA sleep study, representing numbers that are close to their real prevalence in similar populations. A breakdown of those diseases is presented in Supplementary Table S1.

PhysioNet Apple Watch Polysomnography Study

Data for this study was collected at the University of Michigan between 2017 and 2019. The study consisted of 39 healthy subjects with no prior diagnosis of sleep-related breathing disorders, parasomnias, restless leg syndrome, central disorders of hypersomnolence, peripheral vascular disease, cardiovascular disease, vision impairments not correctable by glasses or contact lenses or other disorders that could cause neurological or psychiatric impairment. The study also excluded on the basis of shift work and recent transmeridian travel. Furthermore, participants were ruled out on the basis of excessive daytime sleepiness according to the Epworth Sleepiness Scale, and after the PSG visit, participants which showed symptoms of either obstructive sleep apnoea or REM sleep behaviours were also excluded. A total of 31 subjects met the required criteria. Data for the study can be obtained through Physionet[18] and a detailed description of this dataset is available elsewhere[19].

Participants in this study wore an Apple Watch to collect their activity patterns for 7 to 14 days before spending one night in a sleep lab. During the final night, participants underwent a PSG study while wearing the Apple Watch device (which collected HR and triaxial acceleration). The study was approved by the University of Michigan Review Board and all participants provided written informed consent.

For the PhysioNet Apple Watch study, Apple Watch raw triaxial acceleration data (x, y, z axis measured in g) at a 50Hz resolution was converted into angular postural based metrics like the ones described on BBVS. The Apple Watch measures HR in beats per minute, sampling every several seconds through its PPG sensor. For our analysis, we down-sampled HR by taking the mean of all samples within 15-second windows. For the PhysioNet Apple Watch study, the laboratory technicians started a "recording" period for the watch before the PSG recording started. For our final analysis, we only included participants whose sleep onset and offset were greater than 10 minutes from the start and end of the recording period, respectively. Through this process we intended to introduce a more realistic setting for our model. Details on the laboratory PSG settings can be found elsewhere[19]. The final cohort consisted of 22 participants.

The Multilevel Monitoring of Activity and Sleep in Healthy people (MMASH)

Data for the MMASH study was collected by BioBeats in collaboration with researchers from the University of Pisa and was obtained through Physionet[18,20]. The study collected data from 22 healthy young adult male participants comprising continuous heart rate and triaxial accelerometry monitoring as well as a variety of questionnaires to assess their physical activity, psychological and sleep characteristics as well as a detailed sleep diary. Participants also recorded their perceived mood (Positive and negative Affect Schedule-PANAS), Daily Stress Inventory (DSI) during the free-living protocol and completed a Morningness-Eveningness Questionnaire (MEQ), State-Trait Anxiety Inventory (STAI-Y), Pittsburgh Sleep Quality Questionnaire Index (PSQI) and Behavioural avoidance/inhibition (BIS/BAS) during their clinic visit. Further, anthropomorphic characteristics were recorded. All data was processed and recorded by sport and health scientists with the objective of assessing psychophysiological response to stress stimuli and sleep.

The 22 MMASH participants were fitted with two devices for continuous recording during 2 days: a heart rate monitor (Polar H7, Polar Electro Inc., Bethpage, NY, USA) which recorded beat-to-beat intervals and was used to obtain HR data and a triaxial accelerometer (ActiGraph wGT3X-BT - ActiGraph LLC, Pensacola, FL, USA) was worn on the wrist. Participants were asked to wear the devices continuously during the duration of the protocol and to complete a diary of their sleep onset and wake up times during the recording period. For MMASH we followed the same pre-processing, data quality and noise removal protocols that we described in BBVS for both the triaxial accelerometry signal and the HR signal. Two participants were removed from analysis on the basis of missing diary entries.

All participants provided written informed consent. Information was provided to them regarding the research protocol in accordance with General Data Protection Regulation: Regulation - EU 2016/679 of the European Parliament and of the Council 27/04/2016. Further, all experiments conducted were in accordance with the Helsinki Declaration as revised in 2013, the study was approved by the Ethical Committee of the University of Pisa (#0077455/2018).

Evaluation Details

Evaluation with sleep diary and angle change: BBVS

In the BBVS study, participants wore a variety of wearable devices and recorded the time they went to bed and woke up on a daily basis, providing detailed sleep diaries. As such, we conducted two types of evaluations on this cohort.

Evaluation with sleep diary. First, we compared the performance of our method against those sleep diaries. For our evaluation, we only included participants who had filled out those diaries and had more than three days of concurrent sensing and diary data. We evaluated our model against the diaries in terms of total sleep time, sleep onset and offset.

Evaluation with angle change algorithm. We assessed the performance of our approach versus an angular change algorithm inspired by previous work[10,11]. The angular change approach started with calculating the pitch, roll and z-angle using triaxial acceleration for the device being evaluated. To isolate the gravitational acceleration for each axis, we applied a low-pass filter (0.2 Hertz) to each of the three axes (X, Y and Z) of every recording being evaluated.

Pitch, roll and z-angles were then calculated and the difference between successive epoch values was then smoothed using a 5 minute median rolling window. A threshold method ($< 10^{th}$ percentile of values in that given day $\cdot$ 15) was applied to both columns, dividing the time series into initial sleep and wake blocks.

Of these blocks, only those larger than 30 minutes were kept. Blocks separated by less than 60 minutes were then merged and the largest block was deemed as the main sleep block within the day[11].

Two different angular change evaluations were performed, first, the intersection of the epochs when both pitch and roll calculations agreed on a sleep label created a voting system for a more reliable final sleep window. Alternatively, z-angle only measures were used to generate those sleep metrics as previously described[11]. No significant difference was found when comparing the performance of these two different approaches, so we only report the values obtained from the z-angle measures. All the previous steps were done separately for each limb (dominant and non-dominant wrists, and thigh) on which BBVS participants wore a device.

In BBVS, HR is recorded continuously across the 24-hr period. Thus, the threshold quantile is expected to be lower the longer the sampling interval for the ECDF given that sleep occupies a smaller proportion of the total interval being evaluated.

To evaluate the effect of the chosen ECDF, we analyzed the optimal thresholds and their associated results to better understand how parameter choice may affect the performance of our approach.

Evaluation with polysomnography and sleep diary: MESA.

Evaluation with polysomnography. The recording time for PSG started when the subject's setup was complete, yielding a period of sedentary wakefulness prior to sleep onset. While in an ideal scenario the participant would have been subject to ground truth recording also during the day, this is not a possibility given the nature of PSG. However, this limitation was addressed by evaluating PSG against sleep diary on the same dataset and evaluating our approach against both PSG and diary data. For this evaluation we compared the resulting sleep blocks from PSG, defined as epochs where the participant was in either NREM (N1, N2, N3) or REM sleep, to the sleeping window obtained through our HR algorithm.

Further, in MESA, we explored how our algorithm performed in healthy participants versus participants with sleep disorders. To do so, we first evaluated in the full cohort (n=1,154) and then on the subset of participants with (n=189, 16.4%) and without (n=965, 83.6%) any sleep disorders. The goal of this analysis was to caution and inform about potential limitations that our method may have when evaluating in diseased participants.

Evaluation with sleep diary. PSG derived sleeping windows were compared to sleep diary records in the MESA cohort. This comparison allowed us to further understand the deviations of habitual self-reported sleep to objectively monitored, ground-truth through PSG. For the evaluation we use the same metrics as previously explored in the evaluation against PSG.

Evaluation with polysomnography and angle change: PhysioNet Apple Watch Polysomnography Study.

Evaluation with polysomnography. The PhysioNet Apple Watch study provided a unique opportunity to test our method in a commercial-grade wrist-worn wearable sensor that was concurrently worn during PSG. For this study, we used the same evaluation method explored in MESA, exploring our method based on the night-time concurrent recordings of wearable HR and PSG.

Evaluation with angle change algorithm. Given the multimodal nature of the study, we evaluated both the HR based algorithm and the angular change based algorithm on this population. For this evaluation we followed the same procedure as previously described on BBVS.

Evaluation with sleep diary and angle change: MMASH.

In the MMASH study, participants wore an HR strap and triaxial wrist accelerometer and recorded detailed sleep diaries including the time they fell asleep and woke up, which was filled on a daily basis. For this cohort, we also conducted two types of evaluation following the procedures used during the BBVS evaluation.

Evaluation with sleep diary. First, we compared the performance of our method against the sleep diaries of each participant. We evaluated our approach against the sleep diaries in terms of total sleep time, sleep onset and offset.

Evaluation with angle change algorithm. Similar to our second evaluation in BBVS, we also assessed the performance of our approach against the angular change approach previously described.

Additional Evaluations

Evaluation of traditional algorithms on MMASH

One of the main virtues of the HR algorithm is its ability to detect sleep without requiring sleep diaries while using full-day signals. Traditional methods like the Cole-Kripke[21], Oakley[22], or Scripps Clinic[23] algorithms were not designed to be applied during a full day period and thus perform poorly when applied under those conditions, as observed in Supplementary Table 2. In this table, we observe that the HR method outperforms all algorithms. Upon further evaluation, we found that adding a window to these methods (available in our HypnosPy Python library on GitHub (`https://github.com/HypnosPy/HypnosPy`)) significantly improved their results against their "normal" implementation, although they still fall short when comparing their performance to the HR algorithm in full-day settings.

Table S2. Results for the MMASH dataset. (N = 21) The table compares the results of the HR method against the Oakley[22], Cole-Kripke[21] and Scripps-Clinic[24] method in the MMASH dataset. We also include versions of these algorithms with a window function which was added to boost their performance (versus their normal implementation).

**Sleep param.**	**Metric**	**HR Algo. Full Day - HRD** Value (mean ± 95% CI)	**Oakley Algorithm - Without Min** Value (mean ± 95% CI)	**p-value**	**Oakley Algorithm - With Min 20 min** Value (mean ± 95% CI)	**p-value**	**Cole-Kripke Algorithm - Without Min** Value (mean ± 95% CI)	**p-value**	**Cole-Kripke Algorithm - With Min 20 min** Value (mean ± 95% CI)	**p-value**	**Scripps-clinic Algorithm - Without Min** Value (mean ± 95% CI)	**p-value**	**Scripps-clinic Algorithm - With Min 20 min** Value (mean ± 95% CI)	**p-value**
	Time difference (min)	17.64 ± 47.78	-734.62±39.58	0.00	-46.49±78.90	0.195	-737.22±39.13	0.00	-83.40±77.78	0.048	-734.89±39.89	0.00	-80.34±78.75	0.055
Total sleep time	MSE	0.11± 0.04	0.66±0.03	0.00	0.14±0.05	0.304	0.66±0.03	0.00	0.12±0.05	0.694	0.66±0.03	0.00	0.12±0.05	0.631
	Cohen's kappa	0.75 ± 0.10	0.01±0.00	0.00	0.70±0.09	0.423	0.00±0.00	0.00	0.75±0.10	0.928	0.01±0.00	0.00	0.75±0.09	0.979
Sleep onset	Time difference (min)	-39.14 ± 44.60	579.68±26.64	0.00	21.21±38.41	0.111	581.74±26.36	0.00	35.60±40.63	0.062	580.96±26.91	0.00	35.43±40.61	0.063
Sleep offset	Time difference (min)	-21.50 ± 33.85	-154.94±39.62	0.00	-25.28±63.01	0.894	-155.48±39.45	0.00	-47.81±55.55	0.330	-153.93±39.46	0.00	-44.91±56.46	0.385

Algorithm 1 – Method to estimate sleep periods based on Heart Rate.

A full implementation of this algorithm and a plethora of others is available on the open source Python library HypnosPy at `https://github.com/HypnosPy/HypnosPy/`

Input: W - Wearable Data with Heart Rate Data
Q - Quantile Value (Default: 0.35)
L - Minimal window length (Default: 30)
G - Maximal gap interval in minutes to merge sleep windows (Default: 120)

Output: Sleep window inferences

```
Function HR_Algorithm:
    /* Split data into "experiment days" from 3pm-to-3pm.                              */
    W_D = split_days(W, time=15)
    for d ∈ D do
        /* Extract HR data from wearable device                                         */
        HR = get_HR(W_d)
        /* Calculate quantiles for day                                                  */
        HR^Q = calculate_quantile(HR, Q)
        /* Get sequences that HR < HR^Q                                                 */
        SleepArrays = get_sleep_sequences(HR, HR^Q)
        /* Keep only sequences larger than W.                                           */
        for sleepArray ∈ len(SleepArrays) do
            if lengthInMinutes(sleepArray) < L then
                remove(sleepArray)
        /* Merge Sequences if gap between them is smaller than G                        */
        for i ∈ len(SleepArrays) do
            if get_gap(SleepArray_i, SleepArray_{i+1}) < G then
                merge(SleepArray_i, SleepArray_{i+1})
        /* Select Limits of merged Sleep Window                                         */
        for limit ∈ (onset; offset) do
            Select searchWindow = (limit − 240epochs; limit + 60epochs)
            HR Vol = get_rolling_std_dev(searchWindow, window = 10 epochs)
            From searchWindow select epochs where HRVol ≥ 6 beats per min and add to highVolatilityList
        /* Define final Sleep Window                                                     */
        if onset select last epoch from highVolatilityList then
            Overwrite limit as last epoch
        if offset select first epoch from highVolatilityList then
            Overwrite limit as first epoch
```

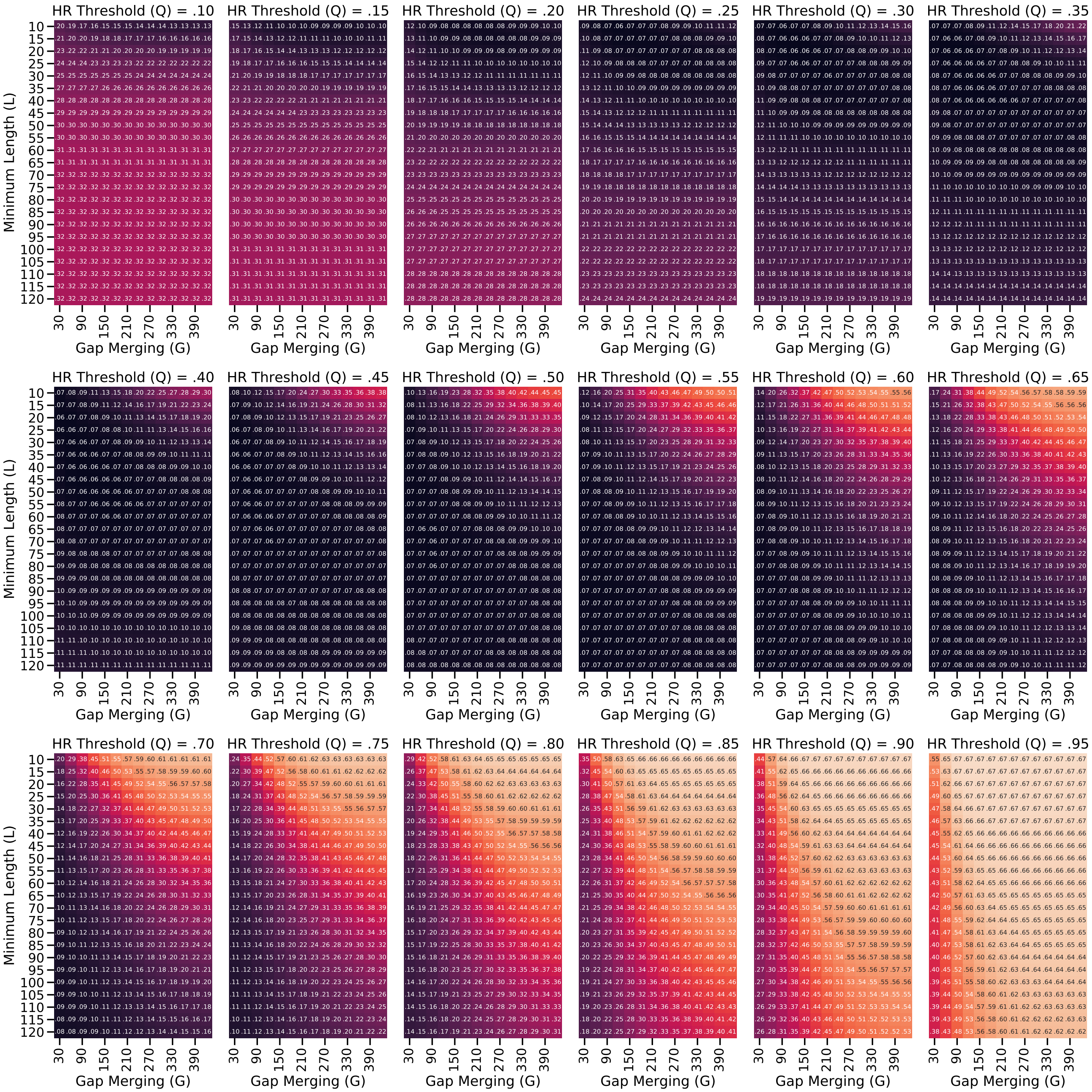

Figure S2. Details of the hyper-parameter search procedure for the full-day HR algorithm on the BBVS dataset.

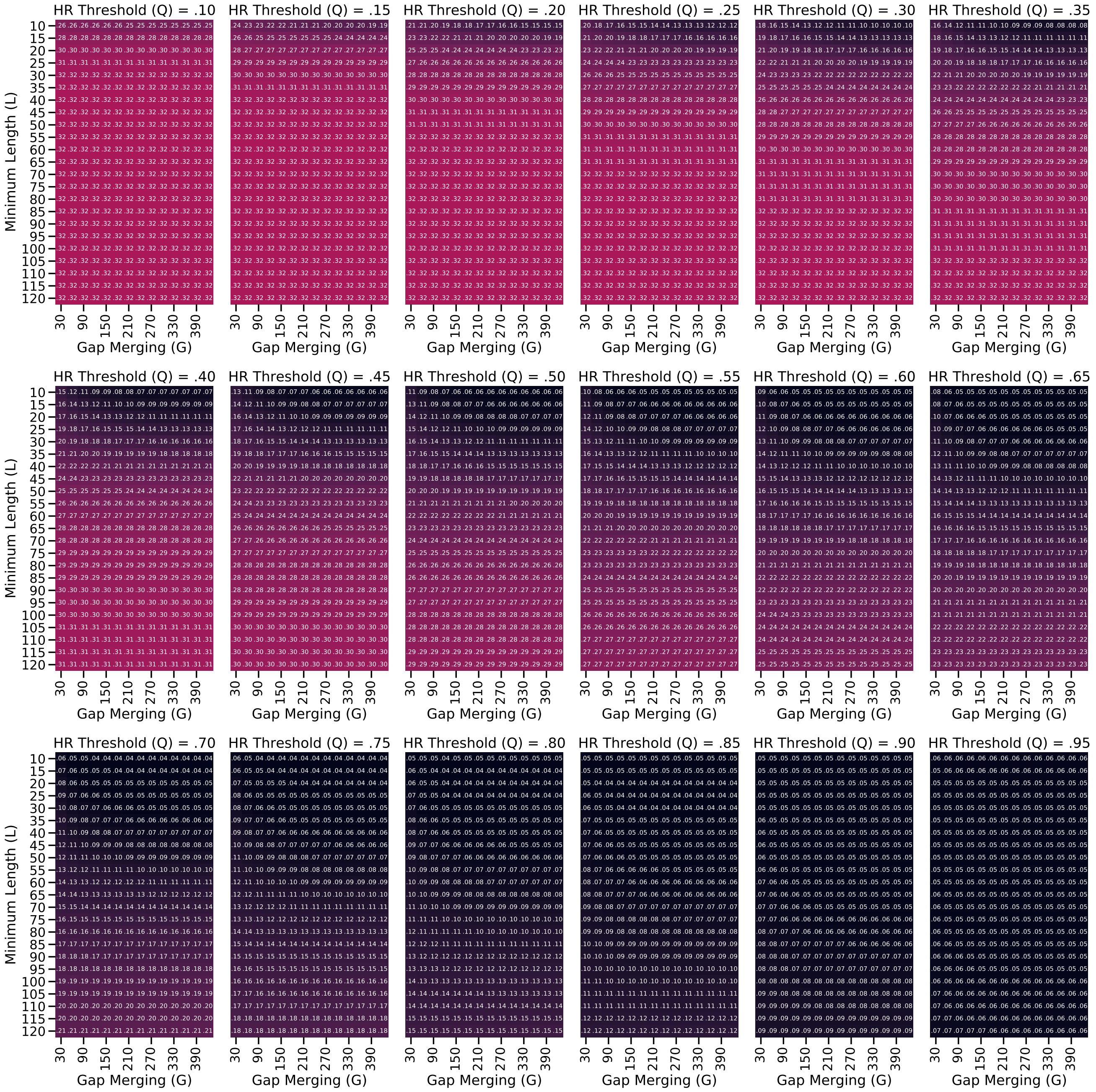

Figure S3. Details of the hyper-parameter search procedure for the night-only HR algorithm on the BBVS dataset.

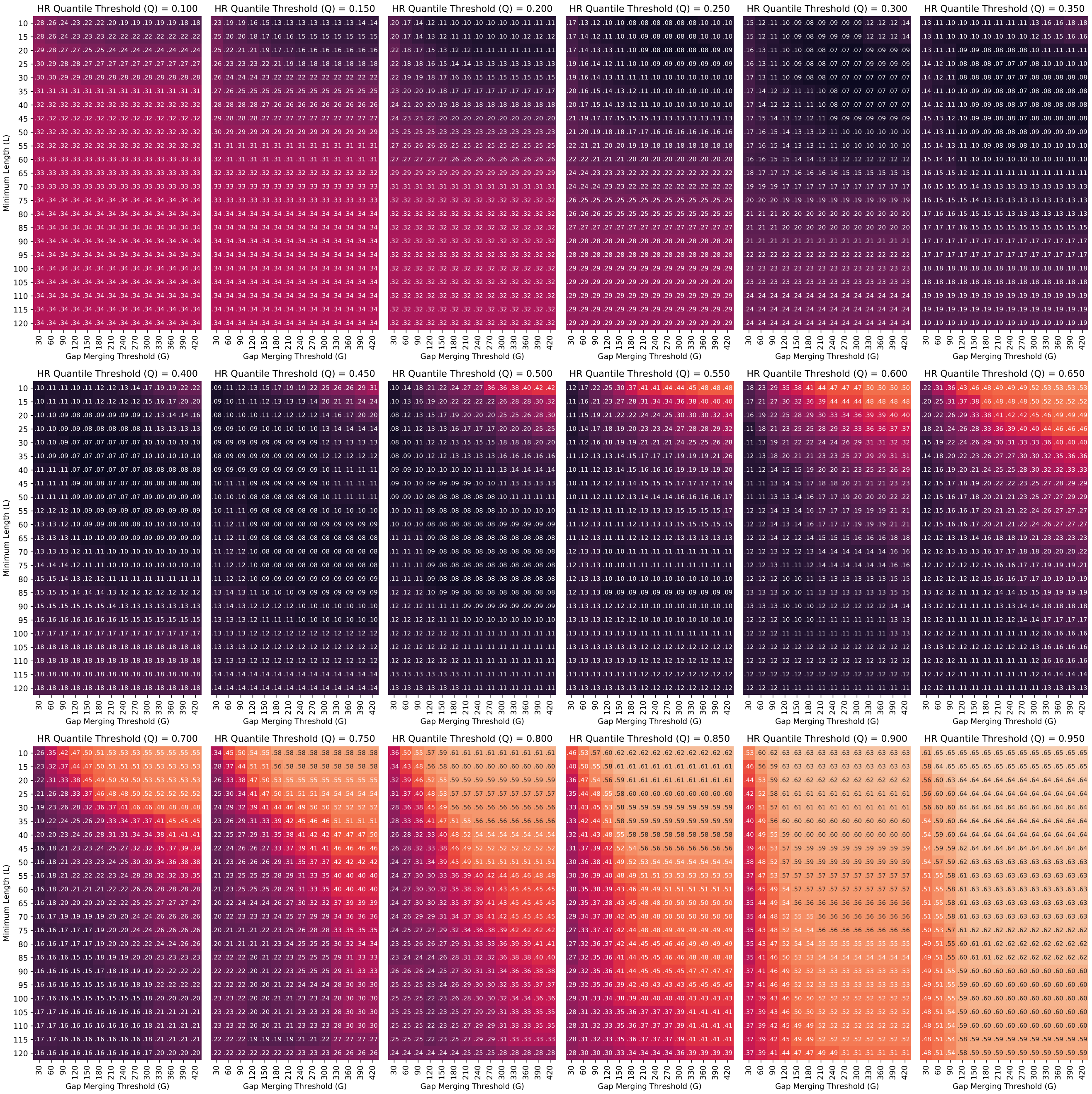

Figure S4. Details of the hyper-parameter search procedure for the full-day HR algorithm on the MMASH dataset.

References

1. Ohayon, M. M., O'Hara, R. & Vitiello, M. V. Epidemiology of restless legs syndrome: a synthesis of the literature. *Sleep medicine reviews* **16**, 283–295 (2012).
2. Roth, T. Insomnia: definition, prevalence, etiology, and consequences. *J. clinical sleep medicine* **3**, S7–S10 (2007).
3. Punjabi, N. M. The epidemiology of adult obstructive sleep apnea. *Proc. Am. Thorac. Soc.* **5**, 136–143 (2008).
4. O'Connor, L., Brage, S., Griffin, S. J., Wareham, N. J. & Forouhi, N. G. The cross-sectional association between snacking behaviour and measures of adiposity: the Fenland Study, UK. *Br. journal nutrition* **114**, 1286–1293 (2015).
5. White, T. *et al.* Estimating energy expenditure from wrist and thigh accelerometry in free-living adults: a doubly labelled water study. *Int. J. Obes.* **43**, 2333–2342 (2019).
6. Brage, S., Brage, N., Franks, P. W., Ekelund, U. & Wareham, N. J. Reliability and validity of the combined heart rate and movement sensor actiheart. *Eur. journal clinical nutrition* **59**, 561–570 (2005).
7. Stegle, O., Fallert, S. V., MacKay, D. J. & Brage, S. Gaussian process robust regression for noisy heart rate data. *IEEE Transactions on Biomed. Eng.* **55**, 2143–2151 (2008).
8. Van Hees, V. T. *et al.* Autocalibration of accelerometer data for free-living physical activity assessment using local gravity and temperature: an evaluation on four continents. *J. Appl. Physiol.* **117**, 738–744 (2014).
9. Lukowicz, P., Junker, H. & Tröster, G. Automatic calibration of body worn acceleration sensors. In *International Conference on Pervasive Computing*, 176–181 (Springer, 2004).
10. Perez-Pozuelo, I. *et al.* Diurnal profiles of physical activity and postures derived from wrist-worn accelerometry in UK adults. *J. for Meas. Phys. Behav.* **1**, 1–11 (2019).
11. van Hees, V. T. *et al.* Estimating sleep parameters using an accelerometer without sleep diary. *Sci. reports* **8**, 1–11 (2018).
12. Dean, D. A. *et al.* Scaling Up Scientific Discovery in Sleep Medicine: The National Sleep Research Resource. *Sleep* **39**, 1151–1164, DOI: 10.5665/sleep.5774 (2016).
13. Zhang, G.-Q. *et al.* The National Sleep Research Resource: towards a sleep data commons. *J. Am. Med. Informatics Assoc.* **25**, 1351–1358, DOI: 10.1093/jamia/ocy064 (2018).
14. Chen, X. *et al.* Racial/ethnic differences in sleep disturbances: the multi-ethnic study of atherosclerosis (mesa). *Sleep* **38**, 877–888 (2015).
15. Varri, A., Kemp, B., Penzel, T. & Schlogl, A. Standards for biomedical signal databases. *IEEE Eng. Medicine Biol. Mag.* **20**, 33–37 (2001).
16. Tanaka, H., Monahan, K. D. & Seals, D. R. Age-predicted maximal heart rate revisited. *J. Am. Coll. Cardiol.* **37**, 153–156, DOI: 10.1016/S0735-1097(00)01054-8 (2001).
17. Malik, M. Heart Rate Variability. *Annals Noninvasive Electrocardiol.* **1**, 151–181, DOI: 10.1111/j.1542-474X.1996.tb00275.x (1996).
18. Goldberger, A. L. *et al.* Physiobank, physiotoolkit, and physionet: components of a new research resource for complex physiologic signals. *circulation* **101**, e215–e220 (2000).
19. Walch, O., Huang, Y., Forger, D. & Goldstein, C. Sleep stage prediction with raw acceleration and photoplethysmography heart rate data derived from a consumer wearable device. *Sleep* **42**, zsz180 (2019).
20. Rossi, A. *et al.* Multilevel monitoring of activity and sleep in healthy people (version 1.0.0). *PhysioNet* (2020).
21. Cole, R. J., Kripke, D. F., Gruen, W., Mullaney, D. J. & Gillin, J. C. Automatic sleep/wake identification from wrist activity. *Sleep* **15**, 461–469 (1992).
22. Oakley, N. Validation with polysomnography of the sleepwatch sleep/wake scoring algorithm used by the actiwatch activity monitoring system. *Bend: Mini Mitter, Camb. Neurotechnology* (1997).
23. Kripke, D. F. *et al.* Wrist actigraphic scoring for sleep laboratory patients: algorithm development. *J. sleep research* **19**, 612–619 (2010).
24. Kripke, D. F. *et al.* Wrist actigraphic scoring for sleep laboratory patients: algorithm development. *J. sleep research* **19**, 612–619 (2010).
